# Theorizing How Context Influences School-Based Interventions That Support Children’s Weight Management: Co-Producing a Refined Logic Model

**DOI:** 10.2196/80309

**Published:** 2026-05-11

**Authors:** Dylan Kneale, Rachael C Edwards, Meena Khatwa, Phuong Tu Nguyen, Sajid Saleem, Sylvia Chaires, Lisa Richardson, Vanessa Bennett, Tabitha Evans, Alison O'Mara-Eves

**Affiliations:** 1 EPPI Centre, UCL Social Research Institute, University College London, C/O: 20 Bedford Way, London, WC1H 0AL, United Kingdom, 44 02076126020; 2 Co-Production Collective, UCL Culture, University College London, London, United Kingdom

**Keywords:** logic model, prioritisation, co-production, children's health, social determinants of health

## Abstract

**Background:**

Schools can play an important role in supporting child weight management, with evidence suggesting that school-based interventions can be effective, albeit with substantial variation. Part of this variation could reflect the different contexts in which interventions are implemented. Our previous work found that co-production of research and adopting a complex systems model of evidence can be transformative for understanding this context. However, less is known about how co-production helps prioritize elements for exploration or how we should assess the value of co-production in research development. This study addresses these gaps by co-producing a refined logic model (a graphical representation of a theory) of the contextual influencers on school-based weight management to support public health research and policymaking.

**Objective:**

This study aimed to (1) identify critical contextual features to consider when planning school-based weight management interventions targeting the intersection of physical activity, healthy eating, and mental well-being; (2) understand the difference that we, as co-producers, make when working together; and (3) identify learning about the process of producing and refining a logic model.

**Methods:**

Through collaborative workshops, we refined an existing theoretical framework identifying contextual influences. We critically reviewed existing frameworks for evaluating co-production, creating a new evaluation approach for our collaborative practices, complemented by surveys and workshop feedback.

**Results:**

Six criteria were identified and applied to refine the original logic model, significantly reducing complexity. The revised model prioritized systemic and policy factors, emphasizing environmental structuring rather than individual behaviors, and incorporated a stronger emphasis on life course factors. We used a tool and process developed specifically for this project, which may have broader utility for other research projects, to understand the value of working together. Many of us recognized that the impacts of the project and working together were unexpected and, for some of us, transformational. Nevertheless, we did identify some factors that could support co-production in future. These included the need for clearer initial discussions on positionalities, role expectations, and autonomy.

**Conclusions:**

Our refined model aligns with broader calls to shift away from narratives centered on personal responsibility in children’s health. This research further demonstrates the practical value and feasibility of co-produced theoretical frameworks, effectively reshaping the focus of research from the outset and challenging established assumptions.

## Introduction

### Schools as a Site of Public Health Action

Children spend approximately a fifth of their waking hours in schools, highlighting the potential of schools as sites that can support children’s health through implementing public health interventions [1]. Reviews across a diversity of topics demonstrate the role that schools can play in supporting children to lead healthier lives [1-4], including the role that schools have in supporting children’s weight management [1,2].

In England, schools play an important role in monitoring the weight status of children, with the annual mandated National Child Measurement Program collecting weight and height data to calculate the BMI of children aged 4‐5 years and 10‐11 years [5]. This measurement program can also serve as a form of intervention to support children’s weight management, with parents being provided with feedback about their children that is designed to help them to correctly recognize their children’s weight status and may stimulate some parents to seek out support [2,6]. More broadly, schools can support children’s weight management through the delivery of universal preventative interventions to promote healthy diets and physical activity, as well as multicomponent lifestyle weight management interventions that support children identified as overweight or obese [7]. While meta-analytic evidence points to schools having an overall positive influence in helping prevent childhood overweight and obesity [8] and supporting weight management [9], estimates point toward high levels of largely unexplained heterogeneity in the success of interventions. Part of this unexplained variation may be attributable to differences in the school context itself, as well as the contexts within which schools are nested. Moreover, despite substantial investment, levels of childhood overweight and obesity in England have broadly stabilized, with over a fifth of children aged 4‐5 years categorized as being overweight or obese and over a third of children aged 10‐11 years [10].

Although children’s weight management is an area where a large evidence base examining the effectiveness of different strategies exists [11,12], there are indications that policymakers tasked with commissioning children’s weight management services do not engage with evidence on the effectiveness of interventions prior to commissioning services [13]. Previous research has indicated that there are missed opportunities for evidence to inform public health decision-making, with public health decision-makers working in local settings expressing an unmet need for contextually sensitive evidence that better reflects the complexity of policymaking decisions [14,15].

This study is part of a larger program that aims to address critical limitations in the way in which evidence is generated to support public health decision-making. Here we report on the development of a co-produced theory about the context surrounding school-based weight management interventions. The focus on context and complexity is congruent with the recognition that school-based interventions supporting childhood weight management take place within systems characterized by multiple factors interacting at different ecological levels [16,17]. Our previous research has also indicated that practitioners and those with lived experience perceive that focusing solely on BMI within the field of children’s weight management, as has been the case within the evidence [8,9], is insufficient to understanding the impacts of interventions and is potentially stigmatizing [18]. Instead, greater prominence should be given to the broader markers that reflect children’s behaviors and capacity regarding weight management [18]. While anthropometric measures such as BMI have a vital role in decision-making and remain fundamental to assessing child growth, there are concerns that the exclusive focus on clinical measures of health could lead children—particularly those with weight management issues—to experience harmful levels of stigma [19]. Therefore, in addition to the focus on the “whole system” of contextual factors, we also theorize how these contextual factors shape the “whole outcome” that is frequently addressed within children’s lifestyle weight management interventions, namely children’s physical activity, healthy eating, and mental well-being.

### Incorporating Complexity and Co-Production in the Way We Create Evidence

The recognition that childhood obesity appears to be largely intractable has prompted the exploration of different approaches around how we think about evidence for children’s weight management. Two key movements of interest here include strategies that apply a systems-based approach to understanding the drivers of (childhood) obesity and approaches that attempt to prioritize people’s lived and professional experience in the conceptualization of complex public health issues and the identification of actions that should be taken.

Evidence that adopts a “complex systems model” recognizes that poor health and health inequalities, such as those observed in the case of childhood obesity, are generated through the interactions of a multitude of interdependent elements within a “system” [20]. For example, children’s weight may be influenced by the actions of teachers or school curricula, which are themselves influenced by the sociopolitical “ecosystem” surrounding children, such as government policies or the actions of large corporations. Taking a complex systems perspective not only involves identifying or theorizing the multifarious individual components included within a system, but also entails recognizing that the emerging health outcomes generated by a system cannot be directly predicted from the elements within it [20]. In the context of childhood health, children and their families have volition, their perceptions, values, and feelings can be unpredictable, and the places in which they live are dynamic.

Alongside calls for an evidence base that reflects the complexity of public health interventions and phenomena is the increasing recognition that academic lenses and perceptions alone are insufficient for unpacking and understanding the complexity of public health issues [21]. There is a growing body of literature demonstrating the value of incorporating people’s lived experience into the design of policies and interventions [22,23]. Co-production is one of a spectrum of approaches to involving the public and patients in the design of research, interventions, and services. The distinctive feature of co-production is the intended formation of equitable partnership with patients, the public, and/or practitioners [24]. Co-production is underpinned by common values (eg, a commitment to challenge the status quo, to being equitable, and to incorporating a diversity of perspectives) and involves working toward a common goal [21,25]. It should be of mutual benefit to researchers and co-producers and seek to reduce traditional power differentials between researchers and research users and beneficiaries [26]. These characteristics can differentiate it from other forms of public participation where, for example, public partners are consulted but have minimal influence over research decisions.

Co-production has been described as adept at capturing the complexity of real-world perspectives and as a research method that “embraces life rather than bracketing it out, neutralizing or controlling it” [27]. While it has been used to design complex public health interventions, there are fewer examples where co-production has been used to theorize complex phenomena. Co-production may be particularly valuable in challenging existing conventions and could help to steer researchers from perpetuating stigmatizing ways of conceptualizing health outcomes. The research we describe here explores how co-production can help us make sense of how health is experienced and how health inequalities form.

### Building on Previous Attempts to Conceptualize Complex Public Health Phenomena and Understand the Role of Co-Production

Previous attempts at understanding the complexity of public phenomena have encountered obstacles. Notably, the Foresight obesity systems map visually sets out the drivers of (adult) obesity through identifying 108 features and 300 connections between the features to depict the factors influencing “energy imbalance” [28]. Although this represented an innovation in the theorizing of obesity, among several critiques leveled at the model were questions about its practical application in supporting theorizing and decision-making, as well as the lack of public involvement in its development [18]. The map depicted a complex system around (adult) obesity but was created through expert and commercial input alone.

Our own preliminary work in this area, where we set out to create a complex system model of the context surrounding school-based weight management interventions, partially addressed some of these critiques through ensuring that the perceptions of parents, teachers, people with lived experience of issues with weight management, public health practitioners, general practitioners, and researchers all occupied a central position in the theorizing. The resulting theory took the form of a logic model, a graphical representation of the different factors and their putative links that influence childhood health. It was created using a co-production and focused on contextual factors that could influence school-based interventions to improve childhood health. This original model included 144 factors organized according to 10 “domains” (types of factors) and across 8 “ecological levels” (see Textbox 1 for more detail on this model). The 144 factors were identified through a series of co-produced workshops and categorized into 10 domains based on common themes and relationships.

Textbox 1. **A description of our starting point—an existing theory of childhood health—the “CEPHI (Co-production of Evidence for Public Health Interventions)” model.**

Our earlier work illuminated the way in which overweight and obesity are conceptualized by stakeholders as markers of a broader set of health challenges facing children and their parents around healthy eating, physical activity, and mental health. Within this project, we co-produced a systems-based logic model [29].

The intention of the original co-produced logic model was to identify contextual features of interest and how they interact to influence school-based interventions. Local authority decision-makers were identified as a key group who could benefit from the model, and it was thus designed with these decision-makers in mind. The model was co-produced with individuals possessing contextually relevant lived and professional experience across a series of workshops. Co-producing this initial model involved up to 13 co-producers located outside the university-based research team, in addition to another 6 university-based co-producers.

**Figure 1.**
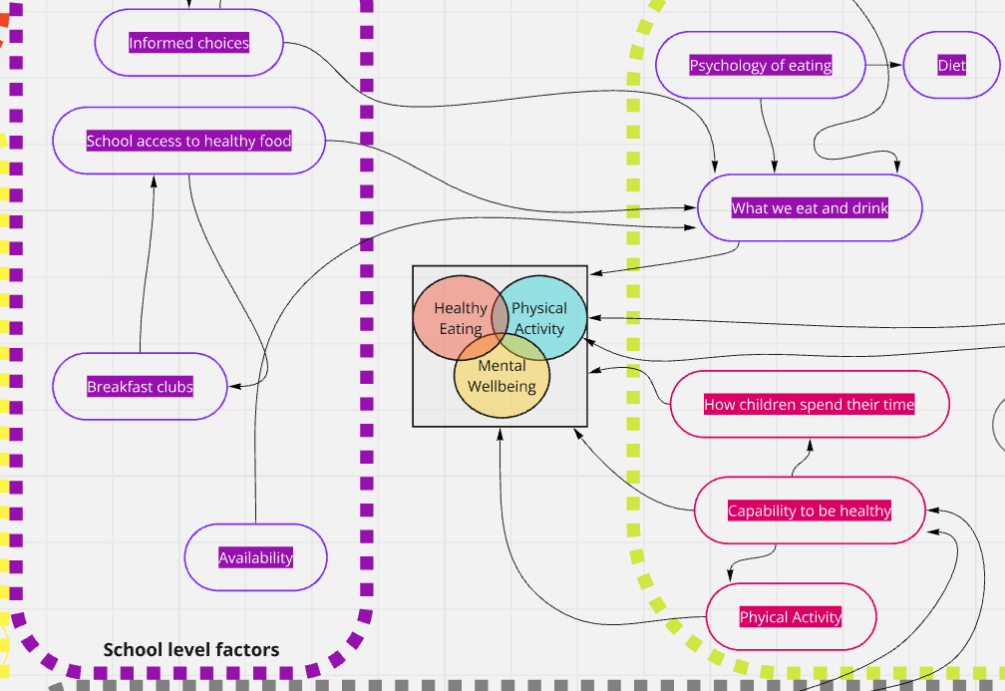
Snapshot of part of the Co-production of Evidence for Public Health Interventions (CEPHI) logic model.

The original logic model consists of almost 150 factors and subfactors that were viewed by co-producers and researchers as important contextual influencers of school-based weight management interventions (with the outcome reflecting the intersection of healthy eating, physical activity, and mental well-being; Figure 1). These factors were split across 10 domains, which represented broad categories of “topics”: media, infrastructure and environment, food, developmental, school, psychological, biological and medical, activity, economic, and “other.” Factors were also split across several ecological levels that influence children’s health from the individual through to a broad cultural level. The model was created using the software Miro (Miro), where one could shift between an overview of the model (snapshot shown in Figure 1) and more detailed depictions of factors and subfactors within each domain.

This array of factors provided a theoretical basis for examining the “whole system” and had an emphasis on socioeconomic and sociopolitical factors [18]. Among the co-production team, this emphasis was viewed as important in shifting the narrative away from one where personal responsibility had previously dominated discourse. Through co-producing the model, the focus of the theorizing switched from a narrow focus on BMI to a more holistic understanding of the components of weight management, namely the intersection of healthy eating, physical activity, and good mental well-being. This “whole outcome” focus also underpins the present research.

Despite the advances that this newer model was perceived to hold over existing models in the literature, the model and the process of its construction raised further analytical concerns. First, questions were raised about the complexity of the model and its practical utility. While depicting almost 150 factors and their relationships appeared to represent a comprehensive theory, the model provided little indication around the relative importance of these factors and where policymakers and practitioners should prioritize their work. Second, we were unsure of how to evaluate the success of the co-production and the extent to which our work had upheld the values of co-production.

The work that is reported here sets out to address these challenges through: (1) co-producing a “whole systems” and “whole outcomes” theory that prioritizes the contextual factors thought to be most influential on children’s levels of healthy eating, physical activity, and mental well-being and (2) developing and piloting an approach to evaluating the success of the co-production. In particular, through building on an existing logic model and refining its contents, the aim of the work is to understand the value of co-production in helping researchers make sense of complex public health issues.

### Aims and Research Questions

This study aims to contribute to developing evidence that better reflects the complexity of the social world, the experiences of people, and the types of questions that arise in public health decision-making [20,30]. Theorizing the features of systems that promote and inhibit health benefits from the perspectives of a wider range of co-producers. However, identifying the optimum ways in which people can come together not only to identify the multifarious features of a system, but also to make decisions about their relative importance, is unclear. Moreover, the optimal way of identifying how and if co-production works in practice and from the perspectives of all co-producers has not been established. This study examines these challenges, and our work was guided by the following research questions:

1. What do we, as a team of co-producers, theorize are the most important contextual features for policymakers, practitioners, and other stakeholders to consider when planning school-based weight management interventions?
2. How can we, as a team of co-producers, understand the difference we make when working together?
3. How did we perceive the way we worked together and what did we learn about the process of producing and refining a theoretical model?

## Methods

### Overall Approaches

#### Overview

This research involved the following approaches:

- To address research question 1, we co-produced a refined theory through a series of workshops and activities to understand the contextual influencers that could help inform the design of policies, interventions, and research, and support the interpretation of evidence on school-based weight management interventions (led by DK and RCE).
- To address research question 2, we undertook a critical review of existing tools to understand their features and developed a new approach to understanding the depth of co-production (led by MK).
- To address research question 3, we evaluated our practice and identified lessons to support future co-production drawing on survey-based and workshop-based approaches (led by LR and VB).

This study is part of a larger study (underway at the time of this writing), and a protocol for the full study is available on the National Institute of Health Research’s Funding and Awards database (see NIHR161485) and registered on the Research Registry (unique identifying number: researchregistry10035). In addition, a protocol for the study addressing research question 2, which took the form of a methodological substudy, is available on the Evidence for Policy and Practice Information (EPPI) Centre website.

The logic model included the emphasis on a “whole outcomes” approach to weight management (the intersection of healthy eating, physical activity, and mental health) that was important to co-producers from different backgrounds. It also is a “whole systems” model that includes considerations from different socioecological contexts, from broad cultural factors to family-level factors [31].

However, while the existing model represents the views of stakeholders, it is also large, complex, and was viewed as unwieldy by some stakeholders (see partial snapshot in Figure 1). The model occupies an uneasy balance between representing some contextual factors of importance (although not all) and can be understood as a partial representation of the complexity of child health with respect to weight management; at the same time, the model is nuanced and complex and does not have a clear starting point for supporting decision-making.

#### Methods for Co-Producing a Refined Theory

Reworking the Co-production of Evidence for Public Health Interventions (CEPHI) logic model involved a series of co-produced workshops in which the initial model was to be refined and involved a core team of 6 members. This core team was comprised of 3 researchers from University College London (UCL) and 3 co-producers based outside UCL (AOME, DK, RCE, PTN, SC, and SS). All co-producers had different blends of relevant lived, living, professional, and practice experience. The co-producers outside UCL were recruited through an open call, and screening conversations were conducted to ensure their availability and commitment to the project. Recruitment happened through UCL’s Co-production Collective, a diverse community of people from a variety of backgrounds who come together to learn, connect, and champion co-production. Joining the workshops was a co-production expert from UCL’s Co-Production Collective who was also an integral team member (VB). Two of the UCL researchers were involved in the creation of the original logic model (AOME and DK).

From April to November 2024, the team engaged in a series of eleven online workshops (totaling 17.5 hours) to refine the existing WOWS logic model for child health. To integrate a greater range of perspectives, we also sought wider stakeholder feedback on an initial draft of the refined model in Autumn 2024 (led by TE). To support our co-production, all co-producers received training in co-production during the first workshop. This training involved familiarizing the co-producers with the values and principles of co-production, clarifying expectations around ways of working and communication, and establishing a shared agreement to guide the collaborative process. The team also co-produced a shared agreement, which helped to clarify expectations around ways of working and communication.

Co-production workshops adopted a semistructured format. UCL-based researchers (DK and RCE) would prepare a loose agenda in advance and act as chair, taking notes and ensuring the workshops progressed. Workshops typically began with a brief presentation from UCL researchers, which would be followed by open discussion. The small size of the team meant that all co-producers typically had the opportunity to feed into each point raised in the discussion. Most workshops were recorded, and following the workshops, EPPI Centre researchers circulated summaries of action points and/or decisions made. All co-producers were encouraged to contribute any additional thoughts or questions between workshops.

Throughout the project, we aimed to enact the values of co-production identified through previous research and by the Co-Production Collective [21,25].

#### Methods for the Development of a Tool to Support Evaluation of Co-Production

This work aimed to:

1. Identify tools and resources that are available to assist in evaluating co-production and understand their features
2. Use the evidence in (1) to inform the co-production of a tool to evaluate co-production of the WOWS project

These aims were met through undertaking a brief review of the most widely applied tools for evaluating co-production. We recognized that tools used to support, report, and evaluate other forms of patient and public involvement may also be useful to consider and included these as appropriate in the review. This brief review itself was not co-produced, although MK, who led this strand, worked closely with the core co-production team and informed their decision-making in developing the evaluation tool. Further details of how the tool was developed are also available elsewhere [32].

#### Methods for Evaluating Our Practice in Co-Production

##### Overview

The third strand of this project involved trialing the new tool and approach for evaluating co-production. This new tool had not been trialed previously, and there were no set guidelines for how it should be administered. This stage of the work involved:

1. Testing the tool itself, developing the approach for administration, and reflecting on this new approach to evaluating co-production, as well as
2. Generating substantive data on how well we perceived that we had worked together and how much the research (in this case, the logic model) reflected all of our input.

LR reviewed the evaluation tool and developed the process for implementing the tool. VB mediated between the core co-production team and LR. LR remained external to the co-production team. LR became a co-evaluator toward the end of the project, analyzing and preparing the data to share with co-producers, observing the final workshop, and leading a reflective workshop.

The evaluation was structured across stages:

##### Stage 1: Individual Summaries and Feedback

All team members started by completing the evaluation tool and LR created individual summaries of our responses. These summaries provided a personalized account of our experiences and perceptions. Each summary included a visual display of our ratings, significant changes in ways of working, and personal reflections on the co-production journey.

##### Stage 2: Individual Sense Check

Each co-producer was then given the opportunity to review their individual summary during this phase. This allowed all members to confirm agreement with the content and make any necessary amendments. Team members could highlight any omissions or request the removal of information they preferred not to disclose.

##### Stage 3**:** Collective Sense-Making

Amended summaries were then integrated into one summary document by LR and shared across the core co-production team prior to a collective sense-making session. This session was critical as it moved the evaluation from individual reflections to developing a collective understanding. Collective sense-making involved a group discussion where key themes and learnings were identified, starting with a visual representation of all individual ratings and working to create a common narrative around how we worked together. A record of this session was then produced and reviewed by the co-production team, a summary of which is reported here.

### Ethical Considerations

This research was conducted following the UK Health Research Authority’s research ethics framework. Ethical approval for the co-production evaluation (REC2046) and stakeholder engagement (REC2053) was granted from the UCL Institute of Education Research Ethics Committee. Informed consent was collected from all co-producers involved in the evaluation of the co-production (but not the stakeholder engagement, as there were no research participants). The consent process was co-produced and was in the form of a shared agreement to which all co-producers signed up. This agreement included that co-producers had the right to withdraw at any time, provisions around anonymity and the right to withdraw direct quotes from being published, and that the decision to be named on the final paper was voluntary. Ethical approval for the creation of the logic model was not needed, as this was conceptual work and there was no research data involved.

## Results

### Describing the Process of Working Together

As a group, we developed criteria around how to prioritize elements of the existing logic model. We started by reviewing the literature on prioritization in public health decision-making, which provided a sense of general categories of criteria that are often applied [33,34]. A few options were presented to the wider team, who were also encouraged to identify additional criteria. After detailed discussion, we decided on a set of 6 questions that were used as a reference when prioritizing the factors and subfactors:

1. Is the feature or pathway likely to impact a large number of children?
2. Does the feature or pathway impact disadvantaged children disproportionately?
3. Is the feature or pathway actionable through local interventions?
4. Could focusing on the feature or pathway stigmatize children and families?
5. Is this a factor that is important because of your experience or the experiences of those you know?
6. Is this a factor that appears most logical to you?

We co-produced a detailed strategy on how to apply these criteria to rank the most and least important features (see Multimedia Appendix 1). We then applied these criteria to each domain to determine if and how the factor and subfactors should be retained and produced an initial draft of the refined model. In addition to the ranking questions, space was also provided within the Qualtrics (Qualtrics) form for us to name any factors we felt were missing, describe the rationale behind our ranking decisions, and name a factor we felt should definitely be retained in the revised model. This latter question was added to ensure that underrepresented perspectives were not lost by only taking the majority view.

### Visualizing the Updated Logic Model

**Figure 2.**
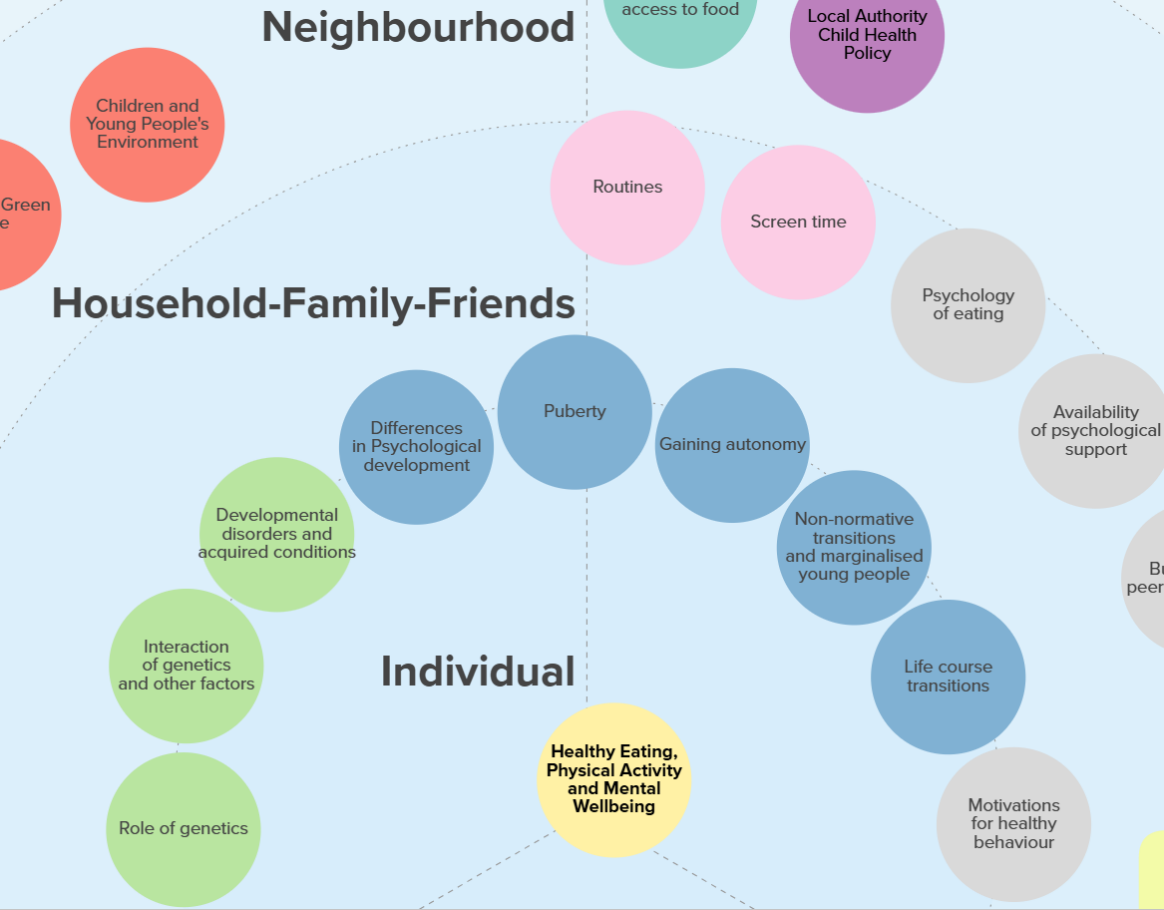
Snapshot of the first draft of the refined logic model in Kumu.

Following the refinement of factors and subfactors in the logic model, EPPI Centre researchers explored how the model could be visualized to be engaging, usable, and accessible. It was decided based on expertise and feedback from the co-producing team that we would use the website Kumu. We drew on the social-ecological model to depict the ecological levels as concentric half circles. The circles broadened from the individual level in the center through sociopolitical and economic levels at the outer edges (Figure 2). The domains were identified through color coding of the factors, which were placed within their corresponding ecological level. Clicking on a factor would bring up a box on the side of the screen containing a detailed description. We chose not to distinguish between factors and subfactors in the updated model.

The refined model was discussed among the co-production team, which provided the opportunity for feedback on both the final list of included factors and subfactors and the visualization. These discussions identified that the omission of the views of children and young people was a weakness, as well as the absence of a wider set of perspectives. It was agreed to explore stakeholder engagement to address these gaps.

### Stakeholder Engagement

#### Overview

As part of our earlier workshops, we identified several relevant voices and perspectives that were missing from within our team. To incorporate these wider perspectives within the model, we invited external stakeholders with lived, professional, and academic expertise to provide feedback on the refined model. We did this in three ways: (1) a blog, (2) engagement with undergraduate students, and (3) further purposive engagement with young people (led by TE).

#### Blog

We wrote a blog (see Multimedia Appendix 2) on the EPPI Centre website explaining the purpose and context of the logic model and requesting anonymous feedback through a free-text box on the blog [35]. We indicated that we were particularly interested in feedback relating to the following questions:

- Which factors are missing?
- Which factors need a better description?
- How holistic is the model?
- How accessible is the model?

We publicized the blog that included a request for feedback through a range of channels, including X (X Corp), LinkedIn (Microsoft Corp), and through email lists.

#### Engagement With Undergraduate Students

As part of a seminar activity for a public health undergraduate module, students were tasked with exploring and discussing the model. We obtained verbal feedback from 2 groups of approximately 15 undergraduate public health students.

#### Purposive Engagement

To ensure that we obtained input from a broader spectrum of young people, one of the wider teams (TE) engaged in informal conversations with young people aged 16‐24 years, discussing the contents of the model and asking for suggestions for improvement.

A final workshop with the co-production team allowed us to reflect on the results of the stakeholder engagement (see Multimedia Appendix 3) and consider if/how suggestions could be incorporated.

### What Do We, as a Team of Co-Producers, Theorize Are the Most Important Contextual Features for Policymakers, Practitioners, and Other Stakeholders to Consider When Planning School-Based Interventions for Improving Children’s Health?

**Figure 3.**
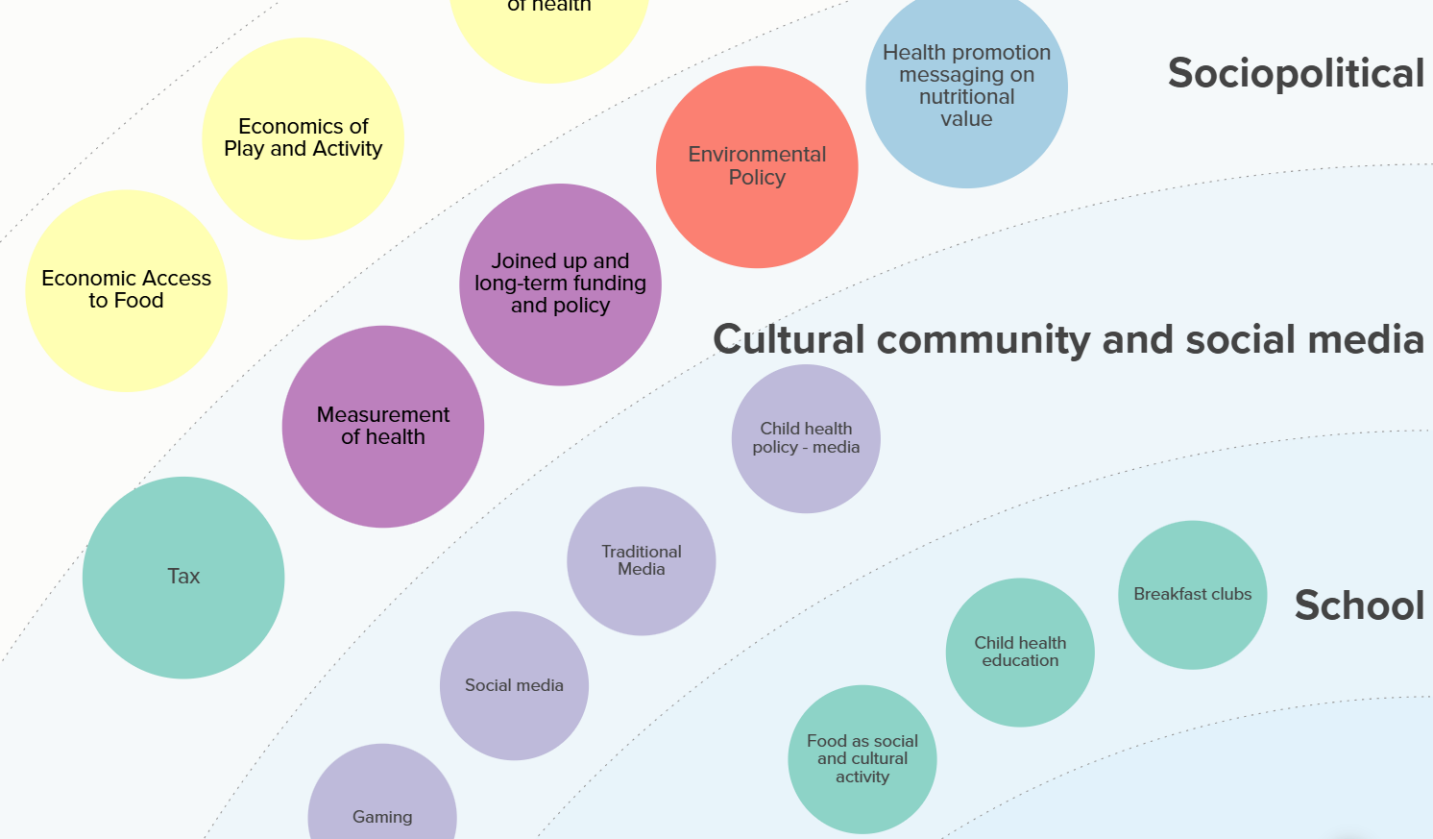
Snapshot of the final draft of the refined logic model.

The new logic model, a snapshot of which is reproduced below (Figure 3) and which can be explored in full here [36], provides a working theory of the 52 factors that influence the whole outcome of healthy eating, physical activity, and mental well-being among children. Across 7 socioecological levels, plus an eighth denoted as “cross-cutting demographic factors,” the model illuminates the factors theorized to influence the outcome and which can either be the subject of an intervention (eg, breakfast clubs) or which may influence the transferability of an intervention effect (eg, features of the economic system including capitalism).

Greater weight is given to the factors that could be considered broader systemic and policy factors that do not focus on changing individual behaviors but are, nevertheless, theorized to influence population health through structuring the environment for health decisions. This greater weight is reflected both in the number of factors, for example, 18 factors are included in the “economic” and “sociopolitical” layers versus 15 factors in the “individual” and “household, family, and friends” layer, as well as the size of the factors (larger in the outer semicircles than the inner semicircles; see Figure 3). These features reflect the narrative of discussions that took place within co-production workshops, which rejected a focus solely on personal or parental responsibility and instead involved greater acknowledgment of the influence of socioeconomic and sociopolitical contextual factors. This means that factors in the food domain, for example, only start to appear at the neighborhood level through the “availability and geographic access to food,” and that previous factors such as the level of energy drink consumption (which had been included within the “individual” level) have been deprioritized and may instead be reflected in policy-level factors such as the “regulation of the food industry.” In this sense, the model theorizes that important contextual influencers of children’s health occur “upstream” at the economic, sociopolitical, and cultural community and social media levels. This is commensurate with classic public health theorizing about the importance of both the social determinants of health and upstream interventions.

The model also helps to identify elements of the context within schools themselves that influence weight management behaviors. For example, these included conscious and unconscious bias and stigmatizing behaviors in the school environment (stigma) and the extent to which schools engage with all children. With respect to the latter, one of our team members reflected that “engagement with [all] children was a particularly sensible choice for me because of the ripple effects that success with this population would have on a generation of minority children and families.”

Our new model also incorporates a greater focus on the life course and development, acknowledging that childhood is a time of flux and shaped by complex interactions between genetics and environment. These factors, rather than behavioral factors, are now represented at the individual level. We also theorize that these transitions—their timing, meaning, and nature—differ by individual characteristics, although they have historically been viewed solely through the lens and norms of nonmarginalized (ie, socially included) people. More broadly, the model acknowledges that the influence of all the factors across the levels is moderated by “cross-cutting demographic factors” including ethnicity, sex, and gender identity.

### What Has Changed in the New Model and How Will It Be Used

The new model offers a more streamlined theory of the system to consider when planning school-based weight management interventions. It has reduced the number of factors included from 144 to 52 (see Table 1) and gives greater weight to factors that are influential outside the individual and household levels. While the influence of upstream factors was acknowledged in the original theorizing, this has been further emphasized in this iteration.

**Table 1.** An overview of the contents of the different domains within the logic model (see text for further information).

Domain name	Number of factors (previous total number of factors and subfactors in brackets)	Number of levels in which the domain is represented (previous number in brackets)
Psychological	9 (25)	4 (5)
Infrastructure and environment	4 (7)	2 (3)
Media	4 (5)	1 (1)
Biological and medical	3 (4)	1 (1)
Activity	3 (14)	1 (6)
Schools	6 (12)	1 (2)
Developmental	5 (3)	1 (1)
Economic	5 (13)	1 (3)
Food	9 (39)	4 (7)
Policy (previously other)	4 (22)	3
All domains	52 (144)	8 (including cross-cutting trends; no change)

Each factor now includes text to support its inclusion, mainly sourced from the co-production discussions (and supplemented where needed), to aid in clarity. Some of the nomenclature has changed, alongside the visualization of the model. Stakeholder engagement informed the way we conceptualized demographic factors so that they were represented as moderating factors at all levels.

Finally, one key change between this model and the previous is the absence of connections between the factors that had indicated the putative causal chains. This omission is deliberate, as the associations between the factors will be investigated and evidenced in later planned work, as well as interventions that could be implemented. However, the size and design of the model do convey the direction of expected effects, with factors at the economic and sociopolitical level viewed as catalysts that influence how factors in layers that are situated closer to the individual are experienced.

### How Can We, as a Team of Co-Producers, Understand the Difference We Make When Working Together?

A more detailed account of the results of the brief review is published elsewhere [32]. Here we focus on the main themes emerging from the review and how these informed the development of elements of the tool developed to evaluate co-production. Based on the analysis of 16 papers and 5 tools, the review identified that existing guidance and resources were more oriented toward supporting the conduct of co-production, embedding it within practice, and reporting co-production, rather than evaluating process or change. Nevertheless, tools and frameworks provided important signals on what could be included when designing a tool to support evaluating co-production, including the need to capture power dynamics, role expectations, and if co-producers felt valued [32]. The themes were used to develop a first iteration of a tool, designed as a self-completion survey, for evaluating the process and influence of co-production. The tool was discussed within co-production workshops and developed further. A third iteration of the tool was used to evaluate the process and influence of co-production in developing the logic model. The full tool, which we refer to through Synthesizing Through Reflection And Participatory Sense-making (STRAPS), is reproduced in [32], and some of the results are presented below.

### How Did We Perceive the Way We Worked Together and What Did We Learn About the Process of Producing and Refining a Theoretical Model?

**Figure 4.**
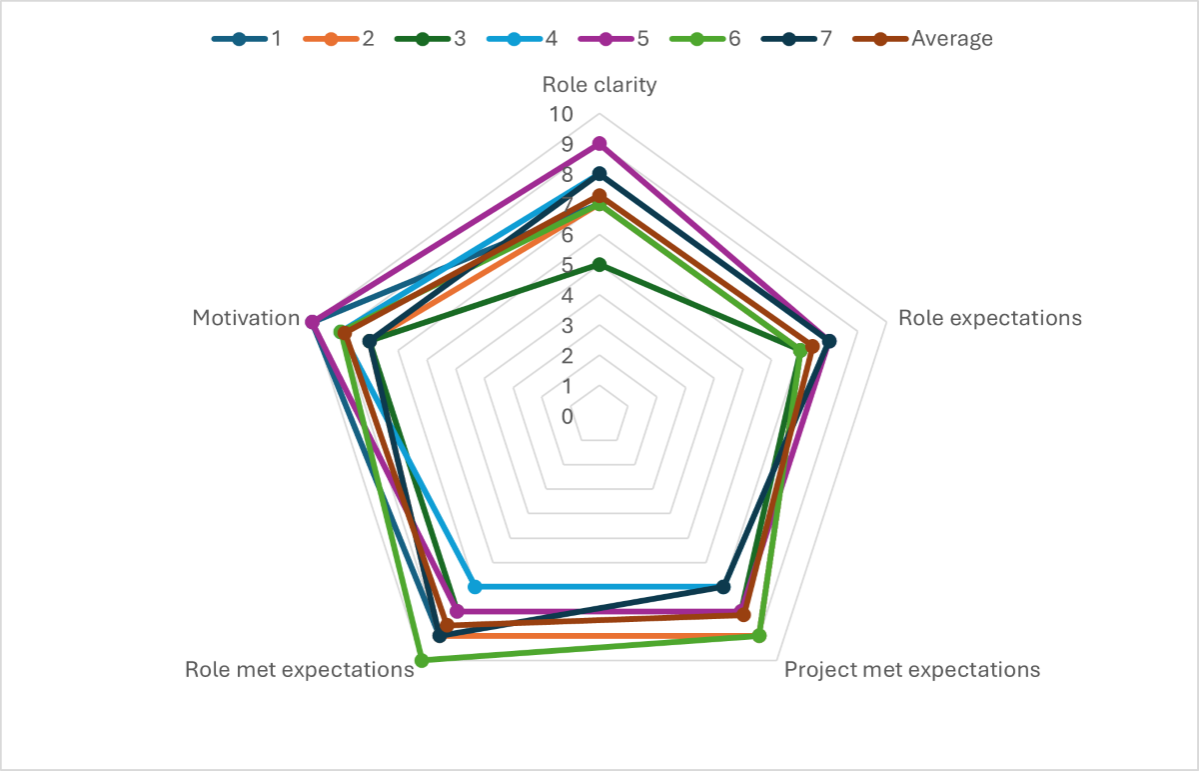
Radar chart showing all team members and average ratings for factors related to role, expectations, and motivation.

The radar chart in Figure 4 shows ratings for items relating to the role expectations, role clarity, and motivation. Scores close to the middle of the chart may indicate a lack of clarity about the role or project or that expectations of the project or role have not been met. For motivation, scores closer to “0” indicate low motivation, and those closer to the edge indicate high motivation (extremely motivated). Role expectations and role clarity showed some variation, with some of us feeling hesitation on what our role was and the expectations on us when the project started. This may also reflect that co-production is a relatively new way of working. Open-ended questions suggested that clarity of role improved over time through the process of working out “what to do and how to do it.” Motivation was high for many of us, and some of us described how working together helped to sustain our motivation. As one of us also reflected, the purpose and end goal of the research itself was also a motivating factor, “the realization that this logic model could inform policy which could then be seen in cafeterias around the country, perhaps in local supermarkets, and neighborhood parks was humbling.” A number of challenges were also raised. These included that a lack of clarity around the role meant we were unclear about how much of our personal or lived experience to contribute to the decisions being made. Another challenge surrounded some of the bureaucratic hurdles we faced (particularly around ethics; see also limitations), which meant that some of the time that we should have taken understanding the processes of co-producing the model itself was instead diverted to understanding and navigating obstacles to the research.

**Figure 5.**
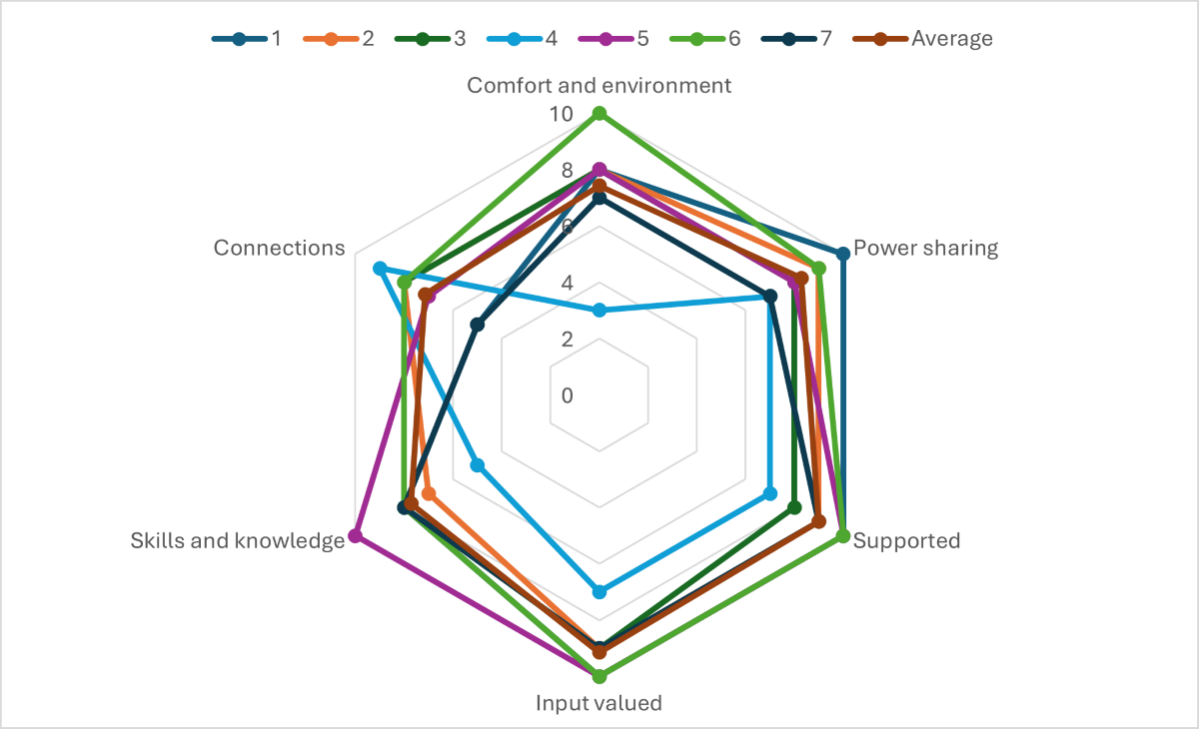
Radar chart showing all team members and average ratings for factors related to co-production processes and outcomes.

We had highly varied scores when it came to assessing the comfort and environment for co-production (Figure 5). Two of the factors that appeared to undermine how comfortable we felt included were (1) uncertainty about how our contributions would “land” with our fellow co-producers and (2) the challenges of working online. Most of us reflected that our contributions were valued and respected and that we developed an “unspoken consensus on equal participation and making all co-producers‘ contributions visible.” For some of us, this was something that evolved over time as we observed that our contributions were valued and as we built relationships, creating something of a virtuous cycle. Not all of us agreed on all points, although “even where there wasn’t consensus there was always validation and acknowledgment of different opinions and a genuine attempt to explore those further.” In addition, it was noted that together we put into place a process for decision-making to prevent minority issues from being voted out by majority view. However, while we adopted an approach where “nothing was predetermined” and all views were considered, some of us observed that sometimes more scaffolding to support decision-making would have been helpful. Finally, although we felt that while we had developed a good “working” relationship, we had missed opportunities to “speak about ourselves in a way that could build a connection beyond our synchronous workshop time.”

### What Were Our Reflections as We Made Sense of Things Together?

Much of our conversation explored the challenge of understanding our positionality (our potential biases and benefits) and thinking about how we share our understanding, views, and experiences.

We acknowledged that social anxieties generally, as well as social preferences, norms, or cultural influences and expectations (eg, not doing small talk, working or living in a different country), have an influence on the way we interact with each other and how we felt at the end of a co-production workshop. Some of us felt that we might want to share more about ourselves in co-production activities in the future, while others of us felt that the process in this project had given us confidence to share personal stories and recognize that “the people around you are not there to judge you, they are, if at all, they can help you, and if nothing else, they can listen.” We recognized that the impacts of the project and working together were unexpected and, for some of us, transformational. This experience helped us to feel more relaxed when communicating with others and encouraged us to think about our relationships more broadly.

We noted the need to overcome pragmatic challenges that could facilitate future exercises and, particularly, some of the difficulties those of us outside UCL faced in claiming compensation for our time. An unresolved challenge we also recognized was the productive tensions that arise in co-production spaces, for example, where academic knowledge conflicts with lived experiences. We questioned how this type of tension can be navigated in the future so that nothing is overlooked and people still feel valued. Some of the places where we did identify some factors that could support co-production in the future were around (1) allowing time for an informal update at the start of meetings that could be personal; (2) considering the possibility of meeting in person, perhaps at the beginning of co-production; (3) building in more discussion at the beginning of co-production around positionalities; and (4) developing and/or communicating role expectations and levels of autonomy more clearly at the beginning of projects.

### How Did the Tool and Evaluation Process Help Our Learning?

We reflected that having more continuous and reflective learning during the project would allow us to implement changes (if any are needed). This can also be seen as part of relationship building, power sharing, and “being human.” However, we also acknowledge that the experience and perceived benefits of co-production are not linear, and in our earlier evaluations, we acknowledged that some of us experienced periods where we felt disconnect or a lack of clarity, which were subsequently resolved. We reflected that the process for administering the tool developed by LR and VB worked well, and most of us liked seeing our collective reflections from our individual reports as a prompt to help us during collective sensemaking. However, we noted that this may not always be the case, and there may not be the same level of comfort in sharing experiences in future projects.

All of our reflections were about the way we created the logic model and not around the logic model itself. This is perhaps further testament to the value of the tool in measuring the process of co-production, but there may be an opportunity to further tease out the perceived value of co-production in the future.

## Discussion

### Summary of Findings

This study aimed to co-produce a logic model that could be used to plan future research. This model would explore which factors influence children’s healthy eating, physical activity, and mental well-being (our whole outcome), with a focus on weight management and school-based interventions. We used a preexisting logic model that had itself been co-produced [18] and aimed here to co-develop this further through establishing a collaboration between co-producers based within and outside UCL. The existing model, while rich and nuanced, was complex and challenging to use to support research and policy in this form. As part of a project taking a “whole outcomes whole systems” approach to evidence, we aimed to explore how further co-production could identify which features should be prioritized in future investigations. The next stages of the research involve us first evidencing the putative pathways through secondary data analysis, and then exploring which interventions may be promising in supporting the promotion of childhood health.

Given that this is a co-produced model, we also wanted to understand how well we had worked together. While we acknowledge that our brief review was not comprehensive (or intended to be), we also observed that many of the available tools, frameworks, and guidelines support users on how to understand and undertake co-production in very specific contexts. Other tools, such as Guidance for Reporting Involvement of Patients and the Public Version 2 (GRIPP2; GRIPP2 Study Group) [37], support the reporting of public involvement in research. Similarly, a number of other tools have been developed to help to evaluate and understand the impact of public involvement more broadly in research [38-40]. However, a gap identified here was in tools that could be used to evaluate the process of co-producing research. While we acknowledge clear overlaps between co-production and other forms of involvement, of particular significance for our needs was the inclusion of questions around power and relational dynamics. The tool proposed and piloted here represents a contribution to addressing this gap for our future projects in the evidence synthesis London Alliance for the Co-production of Evidence Synthesis group. We also questioned whether there was more opportunity to evaluate the difference that co-production makes to the research itself using this kind of approach.

A key output of this research is the generation of a logic model that is the product of a number of different co-producers coming together to identify factors and later prioritize these factors. While created through the input of co-producers with multiple roles and identities, the theorized model embodies a number of the arguments set out earlier around the need to account for complex systems and the need to examine health outcomes holistically. In addition, the model also mirrors calls in the literature to avoid drawing closely on narratives of personal responsibility [41], which perpetuate cultures of stigma around weight and weight management [42], and instead highlight the need to (1) focus more closely on upstream factors and the social determinants of health [43] and the need to (2) consider developmental and demographic characteristics when planning policies and interpreting evidence [44]. Our work illustrates that these are not simply lofty ideals; instead, they are how many people see the world and how they want academics and those in power to see the world. The contribution of the theory developed here is the rejection of these stigmatizing modes of evidence production, although we acknowledge that this theory has not been evidenced at this point.

### Strengths and Limitations

There is emerging familiarity among researchers around the transformative nature of co-production, although there exists uncertainty on how to embed co-production as “normal” research practice [25]. Some of this uncertainty is reflected in the observations made here, for example, the absence of a clear tool to evaluate co-production. However, some of this is also reflected in the limitations we encountered in co-producing this study, including navigating ethical structures. Co-producers of research are not research participants, and conflating co-producers with research participants, for example, by requiring their consent to become researchers, undermines the very endeavor of co-production as a transformative way of working aimed at disrupting traditional power hierarchies in research production. We encountered a number of time delays navigating our own ethical structures, including a requirement to obtain ethical approval for stakeholder engagement, involving the publication of a blog and soliciting anonymous feedback on the logic model. By extension, this could mean that any dissemination activity needs ethical approval. Anxiety around public involvement not only causes delays but also reinforces power hierarchies between those deemed to have the capacity to become researchers and those deemed to lack capacity. While we acknowledge safeguards are needed for vulnerable groups, we question if imposition of a blanket policy could risk undermining future co-production. Certainly, our experience here speaks to the dissonance between institutional ethical review processes and the nuanced realities of undertaking co-produced research [45].

We also note that co-production blurs the distinction between “method” and “result,” particularly as was the case here where the methods themselves are co-produced. Here, the method for prioritizing factors in the logic model was co-produced, as indeed was our whole way of working. This means that we emphasize in our reporting what was decided and how, as might be the case with a process evaluation [46].

UCL co-producers had envisaged up to 6 co-production workshops would take place at the outset, but in practice, the number of co-production workshops and off-line working outside of meetings exceeded this. Such observations could lend support to the concerns leveled elsewhere, which emphasize that the resources that it takes to co-produce research outweigh the benefits [47]. In response, we would emphasize that co-production can take a number of different models [21], and in this study, additional resources brought about significant benefits due to the way prioritization in the model was structured. At its essence, co-production involves academic researchers and those outside academic research working together and agreeing on which decisions will be taken by whom and how. Our chosen model here was to co-produce all decisions, including the number of workshops. On reflection, UCL co-producers acknowledge that this was a highly conceptual project, and the actual number of workshops was commensurate with the challenge of the task. We also reflected that had the project been co-produced from the outset, including co-producing the scope and timelines, then this complexity may have been identified earlier on and been reflected in a greater number of planned workshops.

Two other possible limitations are also worthy of highlighting. The first reflects the number of co-producers, which was lower than had been originally planned. Although stakeholder involvement widened the breadth of perspectives, and the original model had been co-produced with a much larger number of co-producers, this remains a possible limitation. The second is that the model itself does not contain any clearly distinguished causal chains, as might be expected, and even required, to fulfill the criteria of being a “logic model.” While this is a potential limitation, we note that the size and design of the model do convey the direction of expected effects, and that several logic models only give a vague description of the direction of expected effects at the outset of research. Furthermore, these chains are expected to be evidenced further during the course of the project and later work packages.

### Conclusions

As we reflected in the evaluation, this project was highly conceptual and involved co-producing a logic model, which is increasingly recommended within research and evidence syntheses [48]. The work reported here is further testament that this type of practical theorizing can be done with a variety of co-producers, which contributes to changing the focus of the research and challenges the lens of researchers at the outset.

In co-producing this research, our evaluation tool helped us to realize the challenge of bringing our multiple identities to research. The evaluation results also showed that developing clarity around how to work together, our roles in teams, and forming trusting relationships evolved during the project. As was reflected at one point in this work, “being inclusive takes time,” although the clarity that the new logic model brings to supporting the later stages of the research and its potential contribution to informing debates on narratives of personal responsibility in health [49] demonstrates the value of working inclusively through co-production.

## Supplementary material

10.2196/80309 Multimedia Appendix 1 Details of the prioritization approach.

10.2196/80309 Multimedia Appendix 2 Blog to elicit stakeholder feedback.

10.2196/80309 Multimedia Appendix 3 Summary of stakeholder feedback and response.

## Abbreviations

CEPHI: Co-production of Evidence for Public Health Interventions
EPPI: Evidence for Policy and Practice Information
GRIPP2: Guidance for Reporting Involvement of Patients and the Public Version 2
STRAPS: Synthesizing Through Reflection And Participatory Sense-making
UCL: University College London
