# Supplementary material for: Theorizing How Context Influences School-Based Interventions That Support Children’s Weight Management: Co-Producing a Refined Logic Model
Source: J Particip Med. 2026 May 11;18:e80309. doi: 10.2196/80309 (PMC13160485; doi:10.2196/80309)
Supplement: Multimedia Appendix 1 [file jopm-v18-e80309-s001.docx]

## Appendix 1 – Details of prioritisation approach

We decided that each team member would rank all the factors within the existing model individually and these rankings would then be combined across the team. We trialled two ranking approaches but opted for an option involving a ranking question within a survey tool (Qualtrics). We created rankings from most important to the least important, with a limit to the number of factors/sub-factors that could be placed in each ranking to avoid all factors being deemed ‘most important’, with factors ranked in their importance within each individual domain. In addition to the ranking questions, space was also provided within the Qualtrics form for us to name any factors we felt were missing, describe the rationale behind our ranking decisions, and name a factor we felt should definitely be retained in the revised model. This latter question was added to ensure that underrepresented views were not lost when we combined the rankings across team members and took the majority view.

Each factor/sub factor was allocated points depending on its perceived importance. For example, a factor/sub-factor would receive 5 points each time it was ranked highest (most important) box, 4 points for second highest, and so on. The points were then totalled across the team. Each team member ranked all factors and sub factors within each domain. We held drop-in sessions during this period where co-producers could discuss their ranking approach.

After examining the ranking results, we co-produced a process for distinguishing between factors/sub-factor could be a) retained without modification in the refined model b) retained within the description of another factor/sub-factor, or c) removed from the updated model. EPPI Centre researchers then applied these criteria to each domain to determine if/how the factor/sub-factors should be retained and produced an initial draft of the refined model.
