# Supplementary material for: Theorizing How Context Influences School-Based Interventions That Support Children’s Weight Management: Co-Producing a Refined Logic Model
Source: J Particip Med. 2026 May 11;18:e80309. doi: 10.2196/80309 (PMC13160485; doi:10.2196/80309)
Supplement: Multimedia Appendix 2 [file jopm-v18-e80309-s002.docx]

## Appendix 2 – Blog to elicit stakeholder feedback

Our team at the [**London Alliance for the Co-production of Evidence Synthesis (LACES)**](https://eppi.ioe.ac.uk/cms/Default.aspx?tabid=3884) have co-produced a model for child health alongside co-researchers with a range of expertise. This model is a type of theory that depicts the factors thought to impact healthy eating, physical activity, and mental health among young people. The model is focused on **schools** and is designed to **support public health decision makers**.

**We would greatly appreciate you feedback on the model** to enhance its accessibility and value. You can access the model [**here**](https://embed.kumu.io/336ee339b7b1d9d8e73c946ee36c82f0#wows-v1) and provide your comments through the link below.

**Why did we develop this logic model?**

Healthy eating, physical activity, and mental health are complex challenges driven by multiple factors interacting at different levels. An understanding of this complexity is necessary to support public health decision makers in commissioning interventions, and researchers to improve this research. Schools are potentially vital sites for intervening and improving children’s health, and this model helps us to think about which factors may influence a school’s capacity to influence children’s health.

We hope that this model can support the development of holistic **public health policies and programmes relating to schools**. Although it is likely to be of use to many decision makers, we designed the model with **local authority public health teams in mind**. The model could help decision makers consider a range of questions including:

- What are the different channels through which policies and programmes can support child health in schools?
- How might the impact of a school health policy or programme vary across children from different backgrounds and with different life experiences?
- What factors might enable or limit the effectiveness of a school health policy or programme?

**How did we develop this model?**

EPPI Centre researchers and public co-producers with relevant lived/living experience (including parents, teachers, researchers, and young people) previously developed a logic model for child health. We recently worked as a smaller group of co-producers to further refine the model. This smaller co-production team retained a diversity of perspectives – public health researchers, parents, and educational researchers – although we are now seeking more input on this model from a wider group of people.

**How you can help!**

Now it’s your turn! **We’d like your feedback on the model**. We want to hear from anyone who could use and/or benefit from the model including local authority public health staff, nutritionists, teachers, and young adults with lived/living experience of issues around healthy eating, physical activity or mental health. We’re particularly interested in the following questions, but any feedback is welcome:

- Which factors are missing?
- Which factors need a better description?
- How holistic is the model?
- How accessible is the model?

Click [**here**](https://embed.kumu.io/336ee339b7b1d9d8e73c946ee36c82f0#wows-v1) to see the model and provide your comments through the link below:

- Please **do not include identifying information** in your comments
- You must be **at least 18** to provide feedback
- Your **comments won’t be visible** to anyone apart from the research team
- Please contact [embeddedresearchers@ucl.ac.uk](mailto:embeddedresearchers@ucl.ac.uk?subject=Feedback%20on%20Logic%20Model%20draft) if you have any questions about the research
