# Supplementary material for: Theorizing How Context Influences School-Based Interventions That Support Children’s Weight Management: Co-Producing a Refined Logic Model
Source: J Particip Med. 2026 May 11;18:e80309. doi: 10.2196/80309 (PMC13160485; doi:10.2196/80309)
Supplement: Multimedia Appendix 3 [file jopm-v18-e80309-s003.docx]

## Appendix 3 – Summary of Stakeholder Feedback and Response

EPPI Centre researchers presented an overview of the stakeholder feedback to the co-producers through a workshop, along with proposed edits to the model. This was followed by a detailed discussion with co-producers.

Some of the feedback received from stakeholders related to the visual accessibility of the model including the colour scheme and size of the text. There were concerns that the current colour scheme would not be accessible to those with colour blindness and that the labels for the different levels needed to be larger. In response, we are investigating the potential to publish two versions of the model with different colour schemes. Unfortunately, adjusting the size of labels is not possible in Kumu.

Stakeholders also proposed ideas for how the visual dimensions of the model could be adjusted to better communicate conceptual aspects of the model. For example, it was suggested that pictures could be added to the descriptions of factors, that outer factors (i.e., system level factors) could be made larger to better reflect their particularly strong influence on downstream factors, and that factor placement around the model could purposefully cluster those factors with stronger relationships. We plan to implement these changes where Kumu allows.

Stakeholders also identified a wide range of influencers of child health that they felt should be represented as standalone factors. These included cultural influences on diets, the consumption of harmful substances, and health/sexual education in schools. The co-production team carefully considered each suggestion. In most cases, it was decided that these factors could be better reflected in the descriptions of broader factors (which also aligned with the aim of reducing factors in the original model). In a small number of cases, the team created standalone factors based on the stakeholder suggestions.

Stakeholders were also concerned that factor descriptions varied substantially in length and detail. In response, the team decided to create structured abstracts for each factor which would include a one sentence overview, more detailed discussion, and quotes from the team’s ranking data.

Finally, it was felt by stakeholders that some demographic factors were not reflected strongly enough within the model (e.g., sex and gender). The co-production team discussed how demographics were highly relevant across all levels within the model and linked to all factors. As such, it was decided that that demographic factors would be conceptualised within the model as a ray or band crosscutting all levels from the inside to the outside of the model.
